# Combination of baseline and first-week neutrophil-to-lymphocyte ratio predicts overall survival in advanced renal cell carcinoma treated with first-line combination immunotherapy

**DOI:** 10.1007/s10147-026-03115-x

**Published:** 2026-07-11

**Authors:** Takeshi Sasaki, Shinichiro Higashi, Kouhei Nishikawa, Koji Iinuma, Keita Nakane, Tomoki Taniguchi, Yuta Sano, Aika Matsuyama, Kaori Ozawa, Kosuke Tochigi, Masataka Tamura, Yasuaki Kubota, Shusuke Akamatsu, Takuya Koie, Takahiro Inoue

**Affiliations:** 1 Department of Nephro-Urologic Surgery and Andrology, Mie University Graduate School of Medicine, Tsu, Mie Japan; 2 Department of Urology, Gifu University Graduate School of Medicine, Gifu, Japan; 3 Present Address: Department of Urology, Nagoya University Graduate School of Medicine, Nagoya, Aichi Japan; 4 Department of Urology, Japanese Red Cross Nagoya Daiichi Hospital, Nagoya, Aichi Japan; 5 Department of Urology, Gifu Prefectural General Medical Center, Gifu, Japan; 6 Department of Urology, Yokkaichi Municipal Hospital, Yokkaichi, Mie Japan; 7 Department of Urology, Okazaki City Hospital, Okazaki, Aichi Japan; 8 Department of Urology, Toyota Memorial Hospital, Toyota, Aichi Japan; 9 2-174 Edobashi, Tsu, Mie 514-8507 Japan

**Keywords:** Renal cell carcinoma, Neutrophil-to-lymphocyte ratio, Overall survival, Immunotherapy

## Abstract

**Background:**

This study investigated whether neutrophil-to-lymphocyte ratio (NLR) kinetics, including changes within the first week after the initiation of first-line combination immunotherapy, are associated with overall survival (OS) in patients with advanced renal cell carcinoma (aRCC).

**Methods:**

Consecutive patients with aRCC treated with first-line immune checkpoint inhibitor combination therapy at 12 Japanese institutions were included in this retrospective study. The NLR was assessed at baseline (BL), within 7 days (D7), and 1 month (M1) after treatment initiation. Patients were classified into three groups according to NLR at BL and at D7 as follows: BL NLR low (< 3), NLR high-low (BL NLR ≥ 3 and D7 NLR < 3), and NLR high-high (BL NLR ≥ 3 and D7 NLR ≥ 3) groups.

**Results:**

In total, 198 patients were included in the study, with a median follow-up of 18.0 months. The median NLR changed during follow-up at BL, D7, and M1 (BL, 3.28; D7, 3.23; M1, 2.74). OS stratified by NLR at each time point was significant at BL and D7, but not at M1. Among patients with BL NLR ≥ 3 (55%, *n* = 109), 26 patients (24%) had D7 NLR < 3. The 2 year OS rates were 83.7, 85.5, and 66.0% for patients in the low-, high-low-, and high-high groups, respectively. Multivariate analysis revealed that high NLR was an independent risk factor associated with worse OS.

**Conclusions:**

The combination of BL NLR and D7 NLR identified high-risk patients with aRCC treated with first-line immune checkpoint inhibitor therapy.

**Supplementary Information:**

The online version contains supplementary material available at 10.1007/s10147-026-03115-x.

## Introduction

Renal cell carcinoma (RCC) accounts for approximately 2% of all cancers worldwide, and its incidence has consistently increased over the past few decades [1, 2]. Currently, approximately 70% of patients with RCC are diagnosed with early-stage disease (stage I), whereas approximately 11% are diagnosed with advanced or metastatic disease (stage IV) [3]. In patients with early-stage RCC, the 5 year overall survival (OS) rate exceeds 94% after partial or total nephrectomy [3]. As the disease progresses, the average 5 year OS rate gradually worsens: 90% for stage II, 63–78% for stage III, and 28% for stage IV, although individual survival rates vary [3].

In advanced RCC (aRCC), immune checkpoint inhibitors (ICIs) targeting programmed cell death protein 1 (PD-1)/programmed death ligand 1 and cytotoxic T-lymphocyte-associated protein 4 are utilized. First-line combination immunotherapy with ICI + ICI or ICI + tyrosine kinase inhibitor (TKI) has significantly improved the 5 year OS rates compared with those of conventional treatments. For instance, the 5 year OS rate in the CheckMate 214 trial for patients receiving ICI + ICI was 48% [4], whereas the 5 year OS rate in the KEYNOTE-426 trial for patients receiving ICI + TKI was approximately 41.9% [5], indicating substantial improvement.

Prognostic factors for aRCC in the ICI era include conventional stage-related factors, clinical factors included in the International Metastatic Renal Cell Carcinoma Database Consortium (IMDC) risk classification, ICI-era-specific inflammatory and immune biomarkers, pathological factors, tumor immune microenvironment factors, and treatment-related factors, such as immune-related adverse events [4, 6, 7].

In the era of conventional TKI treatment, staging and IMDC risk classification are key factors, whereas the contribution of immune status is limited. By contrast, the host immune response directly determines treatment outcomes in the ICI era. Therefore, inflammation and immune markers are not simply supplementary indicators but also reflect the biological mechanisms underlying the efficacy of ICI treatment.

The neutrophil-to-lymphocyte ratio (NLR) is a representative inflammatory and immune biomarker [7–9]. Neutrophils are generally associated with tumor-promoting effects that enable tumors to evade immune cells, such as lymphocytes, whereas lymphocytes are associated with antitumor immune responses [7, 10, 11]. Therefore, NLR kinetics following ICI administration are considered important, rather than solely baseline (BL) values [12, 13]. A recent meta-analysis of NLR studies, including RCC studies, evaluated the correlation between clinical outcomes and NLR kinetics 2–8 weeks after ICI treatment [14]. However, a recent study reported that the proliferative response of peripheral blood PD-1^+^ CD8^+^ T cells was observed within the first week after the first dose of anti-PD-1 therapy, suggesting that this early expansion is associated with favorable clinical outcomes [15]. Therefore, we hypothesized that NLR within 7 days after ICI administration may be a useful indicator of the balance between neutrophils and lymphocytes in real time, especially in patients with untreated aRCC.

Therefore, the current study aimed to investigate the association between early NLR kinetics, specifically changes within 7 days after the initiation of first-line combination immunotherapy, and OS in a Japanese cohort of patients with aRCC.

## Patients and methods

### Study participants

This multicenter retrospective cohort study was conducted at 12 institutions in Japan, as previously reported [1]. Patients with aRCC who received first-line combination immunotherapy, either ICI + ICI or ICI + TKI, between September 2018 and March 2025 were enrolled. aRCC was defined as metastatic disease detected at the time of RCC diagnosis, disease with postoperative recurrence, or locally advanced cancer that was unresectable. Initial imaging evaluations for aRCC included computed tomography or magnetic resonance imaging of the chest, abdomen, and pelvis. Tumor stage was determined according to the American Joint Committee on Cancer staging system [16]. The ICI + ICI regimen consisted of nivolumab plus ipilimumab, whereas the ICI + TKI regimens included avelumab plus axitinib, pembrolizumab plus axitinib, nivolumab plus cabozantinib, and pembrolizumab plus lenvatinib. This study was approved by the institutional review boards of the participating institutions.

**Fig. 1:**
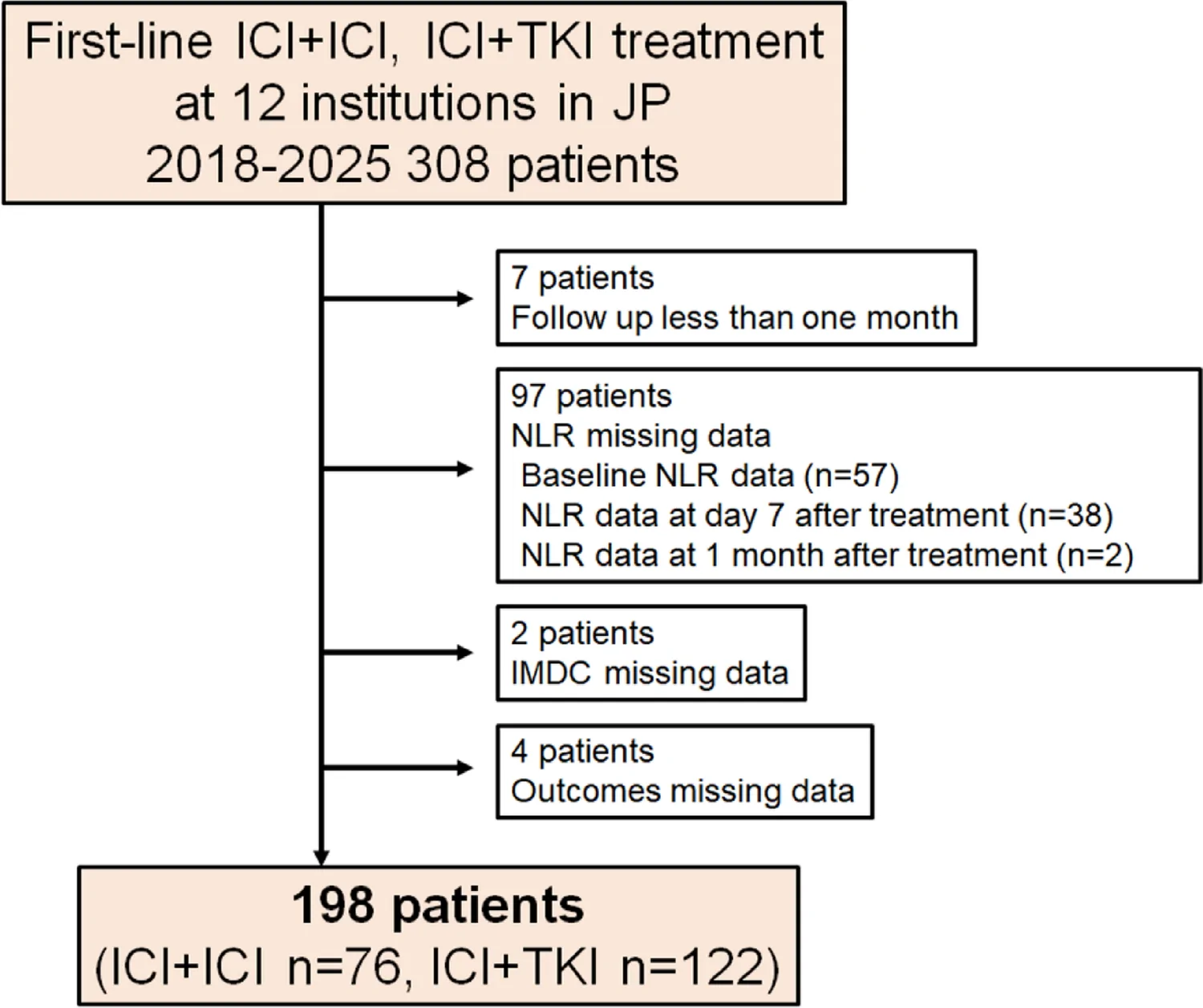
Flow diagram of patient selection

A total of 308 consecutive patients with aRCC were identified. Patients with a follow-up period of less than 1 month (*n* = 7), missing IMDC risk classification data (*n* = 2), or incomplete NLR data at BL or follow-up (*n* = 97) were excluded. Four patients were excluded because of missing outcome data. Finally, 198 patients with aRCC were included in the analysis (ICI + ICI, *n* = 76; ICI + TKI, *n* = 122). A CONSORT diagram is shown in Fig. 1.

Data regarding age, sex, IMDC risk categories, histological type, surgical history, metastatic sites, and treatment agents were also collected. Treatment regimens and dosages were determined at each institution, and treatment was continued until disease progression or unacceptable treatment-related adverse events occurred. The decision to perform nephrectomy, partial resection, or tumor biopsy was also made by the physicians or according to the policies of each institution.

### Follow-up schedule

Patients underwent computed tomography or magnetic resonance imaging every 1–3 months until disease progression. Tumor response was evaluated using the Response Evaluation Criteria in Solid Tumors version 1.1 [17], and the best overall response was classified as progressive disease, stable disease, partial response, or complete response. OS was defined as the time from the initiation of first-line combination immunotherapy to death from any cause.

### NLR kinetics

NLR kinetics were evaluated at three time points: the BL NLR was measured immediately before the initiation of first-line ICI, day 7 (D7) NLR was measured within 7 days after treatment initiation, and 1-month (M1) NLR was measured 1 month after treatment initiation. In all cases, including the patients who had previously undergone nephrectomy, baseline blood test data were collected after the patients had fully recovered from surgery. The specific timing of blood sampling prior to the start of treatment was left to the clinical determination of each institution.

The cutoff value for NLR low (< 3) or high (≥ 3) was set at three based on previous studies [8]. NLR kinetics were classified into the following three groups based on the BL and D7 NLR results: (i) BL NLR low (< 3) group, (ii) NLR high-low group with BL NLR high (≥ 3) and D7 NLR low (< 3), and (iii) NLR high-high group with BL NLR high (≥ 3) and D7 NLR high (≥ 3).

### Statistical analysis

The three groups were compared using the Kruskal–Wallis, Fisher’s exact, and chi-square tests. Longitudinal changes in the NLR were evaluated using the Friedman test. When an overall significant difference was detected, post hoc pairwise comparisons were performed using the Bonferroni correction, with statistical significance set at *P* < 0.016 (0.05/3). OS was evaluated using the Kaplan–Meier method, and intergroup differences were compared using the log-rank test, followed by post hoc pairwise log-rank tests using the Bonferroni correction. Multivariate analysis was performed using a Cox proportional hazards regression model. Multivariate analysis included five explanatory variables because 51 patients experienced a mortality event. Five clinically important variables (IMDC risk, presence of bone metastasis, previous radical or partial nephrectomy, C-reactive protein [CRP] level, and NLR kinetics) were selected based on the existing literature [18, 19] and their medical relevance among accepted potential OS risk factors. Statistical analyses were performed using IBM SPSS Statistics for Windows, version 30 (IBM Corp., Armonk, NY, USA) and EZR version 1.68 [20], a graphical user interface for R (R Foundation for Statistical Computing, Vienna, Austria). *P* < 0.05 was regarded as indicating statistical significance.

## Results

### Patient characteristics

Table 1 summarizes the characteristics of the overall cohort and of patients stratified by NLR kinetics. The median follow-up period was 18.0 months. The median age of the overall cohort was 69.0 years, and 72.7% of patients were men. Among the three groups categorized based on BL NLR and D7 NLR results, significant differences were identified in sex, IMDC risk, histological type, presence of liver metastases, previous renal surgery, BL CRP levels, and BL NLR levels. The high-low NLR group was younger and had a higher proportion of men compared with that of the other groups. By contrast, patients in the high-high NLR group were old, had a high prevalence of metastatic disease, and exhibited high BL CRP and NLR levels.

**Table 1:** Patients’ characteristics (*N* = 198)

Variables		NLR kinetics categorized based on the results of BL NLR and D7 NLR			
	Total cohort, *n* (%)	BL NLR low, *n* (%)	NLR high-low group with BL NLR high (≥ 3) and D7 NLR low (< 3), *n* (%)	NLR high-high group with BL NLR high (≥ 3) and D7 NLR high (≥ 3), *n* (%)	*P* value
No. of patients	198	89 (45)	26 (13)	83 (42)	
Age, Median (IQR)	69.0 (58.6, 73.1)	68.2 (59.0, 73.0)	63.9 (56.5, 72.6)	71.0 (61.0, 74.7)	0.158
*Sex*					0.044
Male	144 (72.7)	58 (65.2)	23 (88.5)	63 (75.9)	
Female	54 (27.3)	31 (34.8)	3 (11.5)	20 (24.1)	
*IMDC risk*					< 0.001
Favorable	28 (14.1)	23 (25.8)	2 (7.7)	3 (3.6)	
Intermediate	117 (59.1)	51 (57.3)	19 (73.1)	47 (56.6)	
Poor	53 (26.8)	15 (16.9)	5 (19.2)	33 (39.8)	
Histology	160 (80.9)	75 (84.3)	24 (92.3)	61 (73.5)	0.008
Clear	16 (8.0)	9 (10.1)	2 (7.7)	5 (6.0)	
Non-clear Not evaluated	22 (11.1)	5 (5.6)	0 (0.0)	17 (20.5)	
*Bone metastasis*					0.057
Yes	49 (24.7)	15 (16.9)	7 (26.9)	27 (32.5)	
No	149 (75.3)	74 (83.1)	19 (73.1)	56 (67.5)	
*Liver metastasis*					0.034
Yes	17 (8.5)	3 (3.4)	2 (7.7)	12 (14.5)	
No	181 (91.5)	86 (96.6)	24 (92.3)	71 (85.5)	
*Prior nephrectomy*					0.007
Radical or partial	62 (31.3)	36 (40.4)	7 (26.9)	19 (22.9)	
Upfront CN	28 (14.1)	17 (19.1)	4 (15.4)	7 (8.4)	
No	108 (54.6)	36 (40.4)	15 (57.7)	57 (68.7)	
*BL CRP (mg/dL)*					< 0.001
Median (IQR)	0.64 [0.15, 3.23]	0.29 [0.08, 1.07]	0.57 [0.11, 3.70]	1.69 [0.48, 7.35]	
*BL NLR*					
Median (IQR)	3.28 [2.20, 4.79]	2.13 [1.69, 2.46]	3.53 [3.30, 4.14]	5.00 [4.07, 6.21]	< 0.001

*IMDC* International metastatic renal cell carcinoma database consortium,* IQR* Interquartile range,* CRP* C-reactive protein,* NLR* Neutrophil-to-lymphocyte ratio,* BL* Baseline, D7, day 7,* CN* Cytoreductive nephrectomy

The sequence of treatment agents stratified by NLR kinetics is shown in Supplemental Table 1. In first-line treatment, the high-low NLR group had a higher proportion of ICI + TKI regimens than that of the other groups. The proportion of patients receiving second-line or later treatment was lowest in the BL NLR low group. Among the 108 patients without prior nephrectomy at baseline, 19 (17.6%) subsequently underwent deferred cytoreductive nephrectomy (CN) after the initiation of combination immunotherapy. The median OS was not reached in the deferred CN group, which was significantly longer than the 43.0 months observed in patients who did not undergo deferred CN (*P* = 0.007) (data not shown).

### NLR and IMDC risk classification

**Fig. 2:**
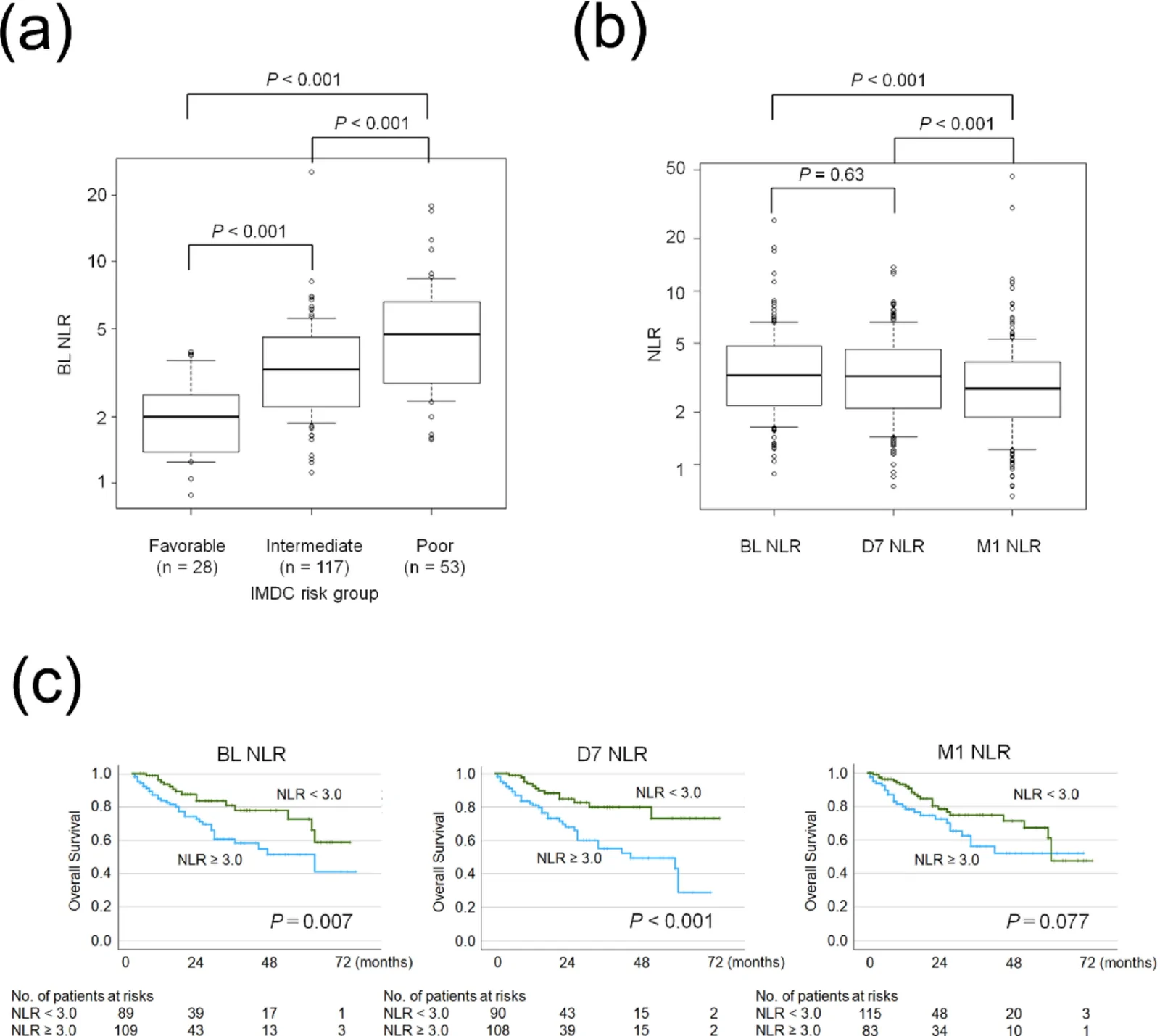
Association between NLR and IMDC risk classification and OS in the overall cohort.** a** NLR and IMDC risk classification;** b** box plot of longitudinal changes in NLR; and** c** longitudinal changes in NLR and OS.* NLR* Neutrophil-to-lymphocyte ratio,* IMDC* International metastatic renal cell carcinoma database consortium,* OS* Overall survival

BL NLR was positively correlated with IMDC risk classification (Fig. 2a). The median BL NLR (interquartile range) for favorable risk (28 patients), intermediate risk (117 patients), and poor risk (53 patients) according to IMDC classification was 2.00 (1.41, 2.48), 3.27 (2.21, 4.58), and 4.73 (2.84, 6.58), respectively, showing significant differences (all *P* < 0.001) (Fig. 2a).

### Longitudinal changes in NLR and OS

NLR in the overall cohort gradually decreased from BL to 1 month after first-line ICI initiation (Fig. 2b). In the overall cohort, the median NLR (interquartile range) at BL, D7, and M1 was 3.28 (2.20, 4.79), 3.23 (2.12, 4.62), and 2.74 (1.87, 3.90), respectively, with M1 NLR significantly lower than BL and D7 NLR (all *P* < 0.001) (Fig. 2b). The Kaplan–Meier curves for OS differed significantly between the NLR low (< 3) and NLR high (≥ 3) groups at BL and D7 (*P* = 0.007 and *P* < 0.001, respectively), whereas no significant difference was observed at M1 (*P* = 0.077) (Fig. 2c).

### Longitudinal changes in NLR low (< 3) and NLR high (≥ 3)

**Fig. 3:**
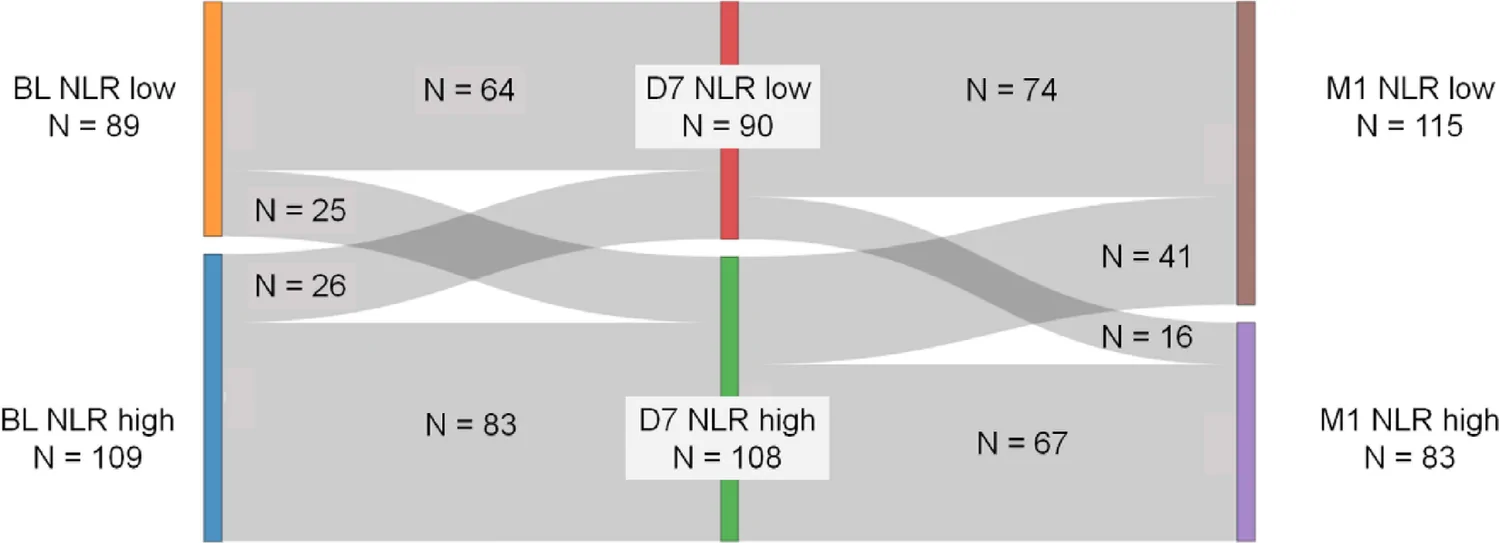
Sankey diagram of longitudinal changes in NLR low (< 3) and NLR high (≥ 3) at three time points (BL, D7, and M1): BL NLR was measured immediately before first-line ICI; D7 NLR was measured within 7 days after treatment initiation; and M1 NLR was measured at 1 month.* BL* Baseline,* D7* Day 7,* M1* 1 Month,* NLR* Neutrophil-to-lymphocyte ratio,* ICI* Immune checkpoint inhibitor

Figure 3a shows the Sankey diagram of longitudinal changes in NLR (Fig. 3). 55% of patients (*n* = 109) had high BL NLR; among these, 26 patients had low NLR at D7. Finally, 58% of patients (*n* = 115) had low NLR at M1 (Fig. 3). In the BL NLR low group, there was no change in absolute neutrophil count (Supplemental Fig. 1a). However, in the BL NLR high group, absolute neutrophil count decreased significantly over time compared with pretreatment levels (Supplemental Fig. 1a). The NLR high-low group demonstrated a significant increase in absolute lymphocyte count (ALC) at D7 (*P* < 0.001), whereas the other groups did not show a significant increase in ALC at D7 (Supplemental Fig. 1b). Furthermore, the longitudinal kinetic patterns of NLR across the three groups in the overall cohort (Supplemental Fig. 2a) were consistent when stratified by the first-line treatment regimen (ICI + ICI vs. ICI + TKI) (Supplemental Fig. 2b and c). In the ICI + ICI cohort, the BL NLR low group exhibited a significant increase in D7 NLR; however, the median value remained within the low-risk range (< 3) (Supplemental Fig. 2b).

### NLR kinetics and oncological outcomes

**Fig. 4:**
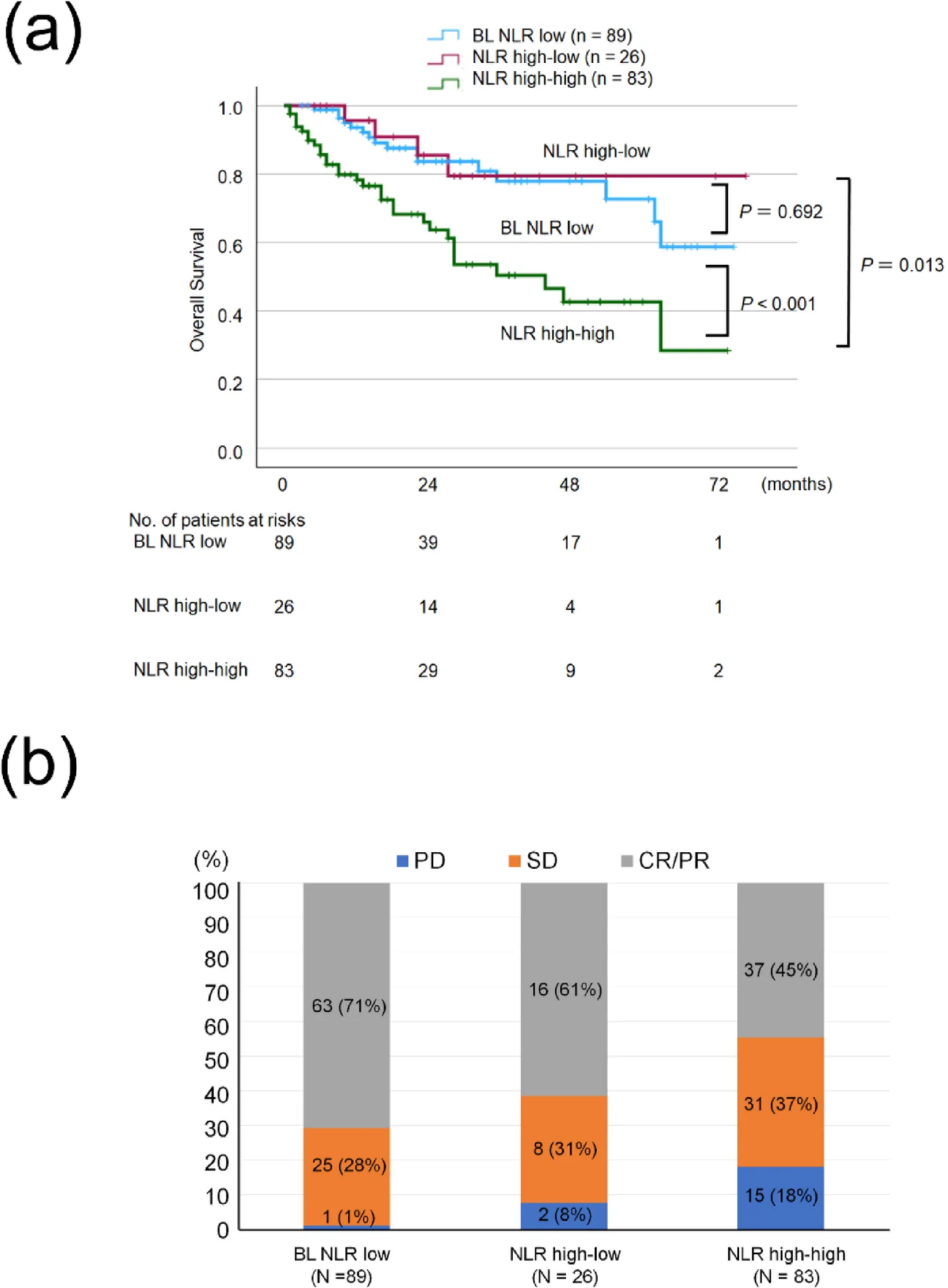
Effects of NLR kinetics on OS in patients with aRCC who received first-line combination immunotherapy.** a** Kaplan–Meier plot of OS and** b** best overall response.* NLR* Neutrophil-to-lymphocyte ratio,* OS* Overall survival,* aRCC* Advanced renal cell carcinoma

In univariate analysis, poor IMDC risk, presence of bone metastasis, absence of prior renal surgery, elevated BL CRP levels (≥ 1.0 mg/dL), and NLR kinetics were significantly associated with worse OS (Table 2). In multivariate analysis, NLR high-high status (adjusted hazard ratio [aHR], 2.04; *P* = 0.03) was an independent risk factor associated with worse OS, whereas IMDC risk classification was not (intermediate risk: aHR, 3.27; *P* = 0.25; poor risk: aHR, 4.89; *P* = 0.14) (Table 2). The 2 year OS rates were 83.7%, 85.5%, and 66.0% for patients in the BL NLR low, NLR high-low, and NLR high-high groups, respectively (Fig. 4a). Pairwise Kaplan–Meier survival curve comparisons between BL NLR low and NLR high-high groups and between NLR high-low and NLR high-high groups revealed significant differences (*P* < 0.001 and *P* = 0.013, respectively) (Fig. 4a). The best overall response demonstrated the highest complete response/partial response rate in the BL NLR low group (71%, 63 patients), whereas the highest progressive disease rate was observed in the NLR high-high group (18%, 15 patients). There was no substantial difference in stable disease rates among the three groups (Fig. 4b). The prognostic significance of these NLR kinetics for OS was maintained when the cohort was stratified by first-line treatment regimens, showing consistent survival trends in both the ICI + ICI and ICI + TKI groups (Supplemental Fig. 3a and b). Although limited by the small sample size (*n* = 7), the ICI + ICI NLR high-low group experienced no mortality events, suggesting an excellent prognosis following rapid NLR normalization (Supplemental Fig. 3a).

**Table 2:** Uni- and multivariate analyses assessing risk factors of OS among patients with advanced RCC who received first-line combination immunotherapy (*N* = 198)

Factor	Univariate	Multivariate
	Hazard ratio [95%CI] (*P* value)	Hazard ratio [95%CI] (*P* value)
Age at which first-line combination immunotherapy was initiated, years (≥ 69 vs. < 69)	1.52 [0.86–2.66] (*P* = 0.14)	–
Sex (female vs. male)	1.36 [0.75–2.46] (*P* = 0.30)	–
*IMDC risk*		
Favorable	1 (Ref.)	1 (Ref.)
Intermediate	5.13 [0.69–37.85] (*P* = 0.10)	3.27 [0.42–25.25] (*P* = 0.25)
Poor	10.00 [1.33–74.82] (*P* = 0.02)	4.89 [0.57–41.85] (*P* = 0.14)
Histology (Non-clear, or not evaluated vs. clear)	0.62 [0.19-2.00] (*P* = 0.42)	–
Bone metastasis (yes vs. no)	1.77 [1.00-3.16] (*P* = 0.05)	1.34 [0.74–2.44] (*P* = 0.32)
Liver metastasis (yes vs. no)	1.02 [0.40–2.57] (*P* = 0.96)	―
Previous radical or partial nephrectomy (no vs. yes)	2.36 [1.30–4.28] (*P* = 0.004)	1.63 [0.82–3.23] (*P* = 0.16)
Treatment regimens (ICI + TKI vs. ICI + ICI)	1.26 [0.70–2.29] (*P* = 0.43)	―
CRP, mg/dL (≥ 1.0 vs. < 1.0)	1.74 [1.00-3.03] (*P* = 0.05)	0.84 [0.42–1.68] (*P* = 0.63)
*NLR kinetics categorized based on the results of BL NLR and D7 NLR*		
BL NLR low	1 (Ref.)	1 (Ref.)
NLR high-low group with BL NLR high (≥ 3) and D7 NLR low (< 3)	0.80 [0.26–2.41] (*P* = 0.70)	0.67 [0.22–2.04] (*P* = 0.48)
NLR high-high group with BL NLR high (≥ 3) and D7 NLR high (≥ 3)	2.85 [1.55–5.23] (*P* < 0.001)	2.04 [1.06–3.92] (*P* = 0.03)

*OS* Overall survival,* IMDC* International metastatic renal cell carcinoma database consortium,* CRP* C-reactive protein,* ICI* Immune checkpoint inhibitor,* TKI* Tyrosine kinase inhibitor,* NLR* Neutrophil-to-lymphocyte ratio,* BL* Baseline, D7, day 7

All hazard ratios represent the ratios of the left-hand-side variables to the right-hand-side variables

Among 109 patients with high BL NLR, 26 achieved rapid normalization within 7 days (Early normalizer), 26 achieved delayed normalization at 1 month (Late normalizer), and 57 remained elevated at 1 month (Non-responder). This distribution of NLR kinetics did not significantly differ between ICI + ICI and ICI + TKI regimens (*P* = 0.378) (Supplemental Table 2). Early normalizers demonstrated significantly better OS than Non-responders (*P* = 0.011), whereas Late normalizers did not (*P* = 0.446) (Supplemental Fig. 4a). In the ICI + ICI subgroup, Early normalizers showed longer OS compared to Late normalizers (*P* = 0.036), but not in the ICI + TKI subgroup (Supplemental Fig.  4b and c).

## Discussion

This study highlights the importance of acute immune monitoring in patients with aRCC treated with first-line ICI. While previous studies have focused on inflammatory markers at 2 weeks or later, we demonstrated that NLR kinetics act as a powerful predictor of survival as early as the first week. The core finding of this study was that rapid normalization of NLR (high to low) within 7 days was driven by systemic recovery of ALC. This finding parallels a recent report indicating that the proliferative response of peripheral blood PD-1 + CD8+ T cells peaks early after the first dose of anti-PD-1 therapy [15]. Therefore, a low NLR pattern within 7 days may serve as a clinical surrogate for immune awakening. To the best of our knowledge, this is the first study to investigate the association between NLR kinetics, specifically changes within 7 days of first-line ICI treatment, and OS in a Japanese cohort of patients with aRCC, demonstrating the prognostic significance of this “ultra-early” immune monitoring.

Among prognostic factors for aRCC in the ICI era, inflammatory and immune biomarkers include NLR, CRP, CRP-to-albumin ratio, lymphocyte-to-monocyte ratio, platelet-to-lymphocyte ratio, systemic immune-inflammation index, and modified Glasgow Prognostic Score [6, 10]. In particular, it is well known that pretreatment NLR values predict oncological outcomes in aRCC [8, 10, 21]; however, studies evaluating post-treatment NLR kinetics remain limited [8, 14]. Table 3 summarizes previous studies on NLR kinetics after first-line ICI treatment in patients with aRCC [11, 18, 22]. For instance, Lalani et al. demonstrated that high NLR at 6 weeks was associated with worse PFS (progression-free survival) and OS [18]. Young et al. demonstrated that NLR decline at 12 weeks in patients with BL elevation (≥ 3) was associated with long OS [11, 22]. A noteworthy aspect of our study is that we evaluated the association between “ultra-early NLR kinetics” and oncological outcomes rather than early NLR kinetics and further assessed longitudinal changes in ALC. Biologically, the significant increase in ALC observed within 7 days in the NLR high-low group may be related to the systemic manifestation of the early proliferative response of effector T cells, which has been reported to occur within the first week of anti-PD-1 therapy [15]. This alignment suggests that the first week serves as a crucial immunological indicator of host transition to an effective antitumor state.

**Table 3:** Studies on NLR kinetics after first-line ICI in patients with aRCC

Study [reference]	Location	Patients number	Cutoff values of NLR	Time points after ICI	Outcomes
Lalani et al. [18]	USA	*n* = 62 (142 cases in the overall cohort)	Decrease ≥ 25%, Increase ≥ 25%	Baseline, 6 (± 2) weeks	PFS, OS
Young et al. [11]	UK	ICI + ICI: *n* = 78; ICI + TKI: *n* = 44	3	Baseline, 12 weeks	Overall response rate, disease control rate, OS
Nakayama et al. [22]	JPN	ICI + ICI: *n* = 84	2.4 at 3 weeks 4.8 at 9 weeks	Baseline, 3, 6, 9 weeks	PFS, OS
Present study	JPN	ICI + ICI: *n* = 76; ICI + TKI: *n* = 122	3	Baseline, day 7, 1 month	Best overall response, OS

*NLR* Neutrophil-to-lymphocyte ratio,* ICI* Immune checkpoint inhibitor,* aRCC* Advanced renal cell carcinoma,* PFS* Progression-free survival,* OS* Overall survival,* USA* United States of America,* UK* United Kingdom,* JP* Japan

Furthermore, comparing the utility of NLR and CRP is clinically important. Recent studies demonstrate that CRP kinetics, such as early normalization or a “flare-response,” strongly predict outcomes in patients with aRCC treated with ICIs [19, 23]. While we lacked post-treatment CRP data to compare kinetics directly in our study, the primary advantage of NLR kinetics over CRP kinetics is its ‘ultra-early’ assessment. Determining CRP kinetics typically requires a 1- to 3-month observation period, whereas D7 NLR provides a much earlier indication of systemic immune response, potentially accelerating clinical decision-making.

Intriguingly, patients whose NLR decreased within 7 days after ICI treatment had a favorable prognosis; in these patients, ALC significantly increased within 7 days after ICI initiation. We speculate that NLR kinetics within 7 days may reflect rapid systemic immune regulation following ICI initiation rather than direct tumor regression. Such changes may represent early rebalancing of circulating immune cells before clinically detectable antitumor effects. Unlike D7 NLR, M1 NLR was not significantly associated with OS in our cohort. This suggests that D7 may capture a relatively pure immunological response before it is confounded by clinical factors, such as immune-related adverse events or rapid tumor progression, which often emerge by 1 month post-treatment [24, 25]. Our supplemental analysis showed that delayed normalization at 1 month yielded inferior survival compared to rapid normalization within 7 days, particularly in the ICI + ICI cohort, reinforcing the clinical necessity of the ultra-early Day 7 evaluation. Biologically, lymphocytes act as a factor that suppresses cancer progression, and their presence within the tumor microenvironment is considered to represent the host immune response [18]. Only two studies have demonstrated an association between low ALC and poor prognosis in patients with aRCC treated with first-line ICI combination therapy [26, 27]. Ueda et al. demonstrated that low BL ALC (< 1289) was an independent prognostic factor for PFS and OS [26], whereas Takemura et al. showed that ALC < 1000 at BL and at 3 months post-treatment was associated with clinical outcomes [27].

Further molecular elucidation of prognostic factors for aRCC in the ICI era is warranted. Several studies have attempted to identify genes that determine responsiveness to ICIs, including BL plasma levels of soluble PD-1, programmed death ligand 1, BTN3A1, and PBRM1 [28, 29]. Furthermore, in the prospective JAVELIN Renal 101 trial of avelumab plus axitinib, pretreatment NLR and gene analyses revealed that NLR values at or above the median were significantly associated with MYC targets and G2-M checkpoint pathways [21]. Thus, a relation may exist between these gene signatures and the NLR kinetics identified in our study.

This study has several limitations. First, this was a multicenter retrospective cohort study with a relatively small sample size, and no central pathological evaluation was performed. Second, pathological findings from biopsy and radical nephrectomy specimens were mixed. Furthermore, pathological confirmation was unavailable for a small number of patients; these patients had typical radiological features of aRCC, and treatment initiation was prioritized based on clinical necessity. Third, the choice of first-line treatment and subsequent second-line or later treatments was determined at the discretion of each institution. Finally, many patients received combination therapy with ICI and TKI, and the potential immunomodulatory effects of TKI on early NLR kinetics cannot be excluded. In the present study, the proportion of patients in the NLR high-low group was slightly higher in the ICI + TKI group (15.5%; 19 of 122 patients) than in the ICI + ICI group (9.2%; seven of 76 patients). This difference may reflect the immunomodulatory effects of TKIs, including suppression of myeloid-derived suppressor cells and tumor-associated neutrophils and reduction of tumor-associated macrophages [30], which could facilitate lymphocyte recovery and attenuation of neutrophil-dominant inflammation, leading to early decline in NLR. However, as demonstrated in our subgroup analyses, both the overall longitudinal patterns of NLR and their prognostic impact on OS were consistent between the ICI + ICI and ICI + TKI cohorts. Furthermore, the treatment regimen itself was not a significant predictor of OS in our analysis, indicating that NLR kinetics is a prognostic factor independent of treatment regimen.

In conclusion, our study demonstrated the prognostic significance of changes in NLR within the first week after initiation of first-line ICI treatment in patients with aRCC. Thus, NLR kinetics may serve as a prognostic biomarker for treatment response and survival outcomes. Clinically, monitoring NLR kinetics as early as the first week enables stratification of patients with a poor prognostic factor (BL NLR ≥ 3) at the outset. Within the NLR high-low group, rapid normalization of NLR suggests an effective immune response, encouraging physicians to confidently continue the current ICI regimen, even when the initial BL NLR is elevated. By contrast, identification of the NLR high-high group serves as a crucial indicator of potential treatment failure, enabling physicians to consider more frequent radiological assessments or earlier transition to subsequent therapies, such as TKIs, to prevent rapid disease progression before standard initial imaging evaluation. Further large-scale prospective studies are required to validate these findings.

## Supplementary Information

Below is the link to the electronic supplementary material.

Supplementary file1 (DOCX 1,809 kb)

Supplementary file2 (XLSX 60 kb)

Supplementary file3 (XLSX 57 kb)

## Data Availability

The datasets generated and analyzed during the current study are available from the corresponding author on reasonable request.
