# Supplementary material for: Combination of baseline and first-week neutrophil-to-lymphocyte ratio predicts overall survival in advanced renal cell carcinoma treated with first-line combination immunotherapy
Source: Int J Clin Oncol. 2026 Jul 11;31(9):1984–94. doi: 10.1007/s10147-026-03115-x (PMC13506512; doi:10.1007/s10147-026-03115-x)
Supplement: Supplementary file 1 [file 10147_2026_3115_MOESM1_ESM.docx]

**Supplemental Figure 1**. Longitudinal changes in absolute neutrophil (a) and lymphocyte (b) counts at three time points (BL, D7, and M1) among the three groups categorized using NLR. BL NLR was measured immediately before first-line ICI, D7 NLR was measured within 7 days after treatment initiation, and M1 NLR was measured at 1 month.

BL, baseline; D7, day 7; M1, 1 month; NLR, neutrophil-to-lymphocyte ratio; ICI, immune checkpoint inhibitor.

**Supplemental Figure 2**. Longitudinal changes in NLR at three time points (BL, D7, and M1) among the three groups categorized using NLR in the (a) overall cohort, and stratified by first-line treatment regimens: (b) ICI + ICI cohort and (c) ICI + TKI cohort.

BL NLR was measured immediately before first-line ICI, D7 NLR was measured within 7 days after treatment initiation, and M1 NLR was measured at 1 month.

NLR, neutrophil-to-lymphocyte ratio; ICI, immune checkpoint inhibitor; TKI, tyrosine kinase inhibitor; BL, baseline; D7, day 7; M1, 1 month.

**Supplemental Figure 3**. Effects of NLR kinetics on OS in patients with aRCC who received first-line combination immunotherapy stratified by first-line treatment regimens: (a) Kaplan-Meier plot of the ICI + ICI cohort and (b) Kaplan-Meier plot of the ICI + TKI cohort.

NLR, neutrophil-to-lymphocyte ratio; OS, overall survival; ICI, immune checkpoint inhibitor; TKI, tyrosine kinase inhibitor.

**Supplemental Figure 4**. Kaplan-Meier OS curves according to 1-month NLR kinetic patterns among the BL NLR high (≥ 3) group: (a) overall cohort, (b) ICI + ICI cohort, and (c) ICI + TKI cohort.

NLR kinetic patterns are classified as Early normalizer (within 7 days), Late normalizer (at 1 month), and Non-responder.

NLR, neutrophil-to-lymphocyte ratio; OS, overall survival; BL, baseline; ICI, immune checkpoint inhibitor; TKI, tyrosine kinase inhibitor.
