# Supplementary material for: Combination of baseline and first-week neutrophil-to-lymphocyte ratio predicts overall survival in advanced renal cell carcinoma treated with first-line combination immunotherapy
Source: Int J Clin Oncol. 2026 Jul 11;31(9):1984–94. doi: 10.1007/s10147-026-03115-x (PMC13506512; doi:10.1007/s10147-026-03115-x)
Supplement: Supplementary file 2 [file 10147_2026_3115_MOESM2_ESM.doc]

| **Supplemental Table 1.** Therapeutic agents | | | |
| --- | --- | --- | --- |
|  |  |  |  |
| NLR kinetics categorized based on the results of BL NLR and D7 NLR (n) | 1^st^ line treatment % (n) | Proportion of patients receiving 2^nd^ line or later treatments % (n) | Detailed information on drugs for 2^nd^ line treatment and later % (n) |
| BL NLR low (89) | ICI + ICI 38.2 (34)  ICI + TKI 61.8 (55) | 29.2 (26/89) | ICI 3.3 (3)  TKI 39.3 (35)  mTORi 1.1 (1) |
| NLR high-low group with BL NLR high (≥ 3) and D7 NLR low (< 3) (26) | ICI + ICI 26.9 (7)  ICI + TKI 73.1 (19) | 42.3 (11/26) | ICI 7.7 (2)  TKI 61.5 (16)  mTORi 3.8 (1) |
| NLR high-high group with BL NLR high (≥ 3) and D7 NLR high (≥ 3) (83) | ICI + ICI 42.2 (35)  ICI + TKI 57.8 (48) | 38.5 (32/83) | ICI 3.6 (3)  TKI 51.8 (43)  mTORi 6.0 (5) |
| Abbreviations |  |  | |
| NLR, neutrophil-to-lymphocyte ratio; BL, baseline; D7, day 7; ICI, immune checkpoint inhibitor; TKI, tyrosine kinase inhibitor; mTORi, mammalian target of rapamycin inhibitors | | | |
