# Supplementary material for: Combination of baseline and first-week neutrophil-to-lymphocyte ratio predicts overall survival in advanced renal cell carcinoma treated with first-line combination immunotherapy
Source: Int J Clin Oncol. 2026 Jul 11;31(9):1984–94. doi: 10.1007/s10147-026-03115-x (PMC13506512; doi:10.1007/s10147-026-03115-x)
Supplement: Supplementary file 3 [file 10147_2026_3115_MOESM3_ESM.doc]

| **Supplemental Table 2.** Comparison of NLR normalization patterns by treatment regimen among high BL NLR (≥ 3) patients | | | |
| --- | --- | --- | --- |
|  |  |  |  |
| NLR kinetics | ICI + ICI (n=42) | ICI + TKI (n=67) | *P* value |
| Early normalizer  [D7 NLR low (< 3)] | 7 (16.7%) | 19 (28.4%) | 0.378 |
| Late normalizer  [D7 NLR high (≥ 3), and M1 NLR low (< 3)] | 11 (26.2%) | 15 (22.4%) |  |
| Non-responder  [M1 NLR high (≥ 3)] | 24 (57.1%) | 33 (49.3%) |  |
| Abbreviations |  |  | |
| NLR, neutrophil-to-lymphocyte ratio; BL, baseline; D7, day 7; M1, 1 month; ICI, immune checkpoint inhibitor; TKI, tyrosine kinase inhibitor | | | |
